# Statistical Study of Low-Intensity Single-Molecule Recognition Events Using DeepTip^TM^ Probes: Application to the Pru p 3-Phytosphingosine System

**DOI:** 10.3390/biomimetics8080595

**Published:** 2023-12-08

**Authors:** Rafael Daza, María Garrido-Arandia, Daniel Corregidor-Ortiz, Carla Isabel Pérez, Luis Colchero, Raquel Tabraue-Rubio, Manuel Elices, Gustavo V. Guinea, Araceli Diaz-Perales, José Pérez-Rigueiro

**Affiliations:** 1 Departamento de Ciencia de Materiales, ETSI Caminos, Canales y Puertos, Universidad Politécnica de Madrid, 28040 Madrid, Spain; rafael.daza@upm.es (R.D.); daniel.corregidor@ctb.upm.es (D.C.-O.); carlaisabel.perez.cova@alumnos.upm.es (C.I.P.); m.elices@upm.es (M.E.); gustavovictor.guinea@ctb.upm.es (G.V.G.); 2 Center for Biomedical Technology (CTB), Universidad Politécnica de Madrid, Pozuelo de Alarcón, 28223 Madrid, Spain; 3 Centro de Biotecnología y Genómica de Plantas (CBGP), Instituto Nacional de Investigación y Tecnología Agraria y Alimentaria (INIA/CSIC), Universidad Politécnica de Madrid (UPM), 28223 Madrid, Spain; maria.garrido@upm.es (M.G.-A.); araceli.diaz@upm.es (A.D.-P.); 4 Departamento de Biotecnología-Biología Vegetal, Escuela Técnica Superior de Ingeniería Agronómica, Alimentaria y de Biosistemas, Universidad Politécnica de Madrid (UPM), 28040 Madrid, Spain; 5 Bioactive Surfaces S.L., C/Puerto de Navacerrada 18, 28260 Galapagar, Spain; luis.colchero@ctb.upm.es; 6 Biomedical Research Networking Center in Bioengineering, Biomaterials and Nanomedicine (CIBER-BBN), 28029 Madrid, Spain; 7 Biomaterials and Regenerative Medicine Group, Instituto de Investigación Sanitaria del Hospital Clínico San Carlos (IdISSC), C/Prof. Martín Lagos s/n, 28040 Madrid, Spain

**Keywords:** affinity atomic force microscopy, force spectroscopy, AFM, functionalization, phytosphingosine, lipid transfer protein

## Abstract

The interaction between the plant lipid transfer protein Pru p 3 and phytosphingosine was assessed using an atomic force microscope. Phytosphingosine was covalently immobilized on DeepTip^TM^ probes and Pru p 3 on MicroDeck^TM^ functionalized substrates. Single-molecular interaction events between both molecules were retrieved and classified and the distribution for each one of the identified types was calculated. A success rate of over 70% was found by comparing the number of specific Pru p 3-phytosphingosine interaction events with the total number of recorded curves. The analysis of the distribution established among the various types of curves was further pursued to distinguish between those curves that can mainly be used for assessing the recognition between phytosphingosine (sensor molecule) and Pru p 3 (target molecule) in the context of affinity atomic force microscopy, and those that entail details of the interaction and might be employed in the context of force spectroscopy. The successful application of these functionalized probes and substrates to the characterization of the low-intensity hydrophobic interaction characteristic of this system is a clear indication of the potential of exploiting this approach with an extremely wide range of different biological molecules of interest. The possibility of characterizing molecular assembly events with single-molecule resolution offers an advantageous procedure to plough into the field of molecular biomimetics.

## 1. Introduction

Although the principles of biomimetics [1] may be applied to systems that comprise all length scales typically found in nature, there is a growing interest in exploring specifically those systems whose properties depend on the details of their organization and micromechanisms at a molecular level. The creation of artificial muscles [2] and of new adhesives based on the mussel attachment mechanism to a substrate [3] are just two examples of the possibilities offered by this field. Consequently, there is an increasing pressure to develop characterization procedures that allow the determination of the properties of these systems and, in particular, of the interactions between their constituents at a molecular scale. In this work, it is shown how atomic force microscopy (AFM) [4] may be used to characterize the interaction between two biomolecules: the lipid transfer protein (LTP), Pru p 3, and its ligand, phytosphingosine.

Compared with other microscopies, AFM offers a number of suitable features for the study of soft matter systems [5], including biological systems [6,7]. Among the singularities offered by AFM should be highlighted the possibility of analysing samples with nanometre spatial resolution [8], while working with living cells under physiological conditions [9]. Furthermore, since AFM is based on the direct contact of the probe with the surface of interest, it is possible to manipulate the system, such as in the experiments based on the analysis of the behaviour of a cell under compression [10].

The potential of AFM is considerably increased by the possibility of attaching a sensor molecule to the probe [11,12]. In this case, the interaction between the AFM probe and the sample results in a specific event that may provide detailed information on the presence of the target molecule in an area of interest [13,14] or on the proper interaction of sensor and target molecules [15]. The first perspective leads to the so-called affinity (or chemical) atomic force microscopy (A-AFM) [11,16] while the latter is referred to as force spectroscopy [17].

However, the implementation of any of these characterization procedures depends critically on a purely technical detail: the AFM probe must be functionalized so that the sensor molecule can be immobilized on its surface. The functionalization process and the subsequent decoration of the AFM probe with the sensor molecule must be the result of a sufficiently robust and versatile procedure. Often, functionalization is performed following a handcrafted process based on a few protocols [18,19,20,21,22] that tend to present difficulties in terms of reproducibility when translated between different laboratories.

DeepTip^TM^ functionalized AFM probes [23] have shown a number of advantages that allow the overcoming of the previously mentioned drawbacks. To begin with, DeepTip^TM^ probes present reproducible features in terms of the surface topography and surface density of the reactive amine groups [24]. In addition, DeepTip^TM^ probes are compatible with a wide range of sensor molecules through varied crosslinking chemistries, and allow the identification of single-molecule recognition events. These properties of the DeepTip^TM^ probes lead to a high success rate of molecular recognition events that may reach over 80% of the total number of recorded curves [24], in comparison with the usual values in the range of 1% commonly reported [25,26]. However, since all these desirable features were exhibited using the streptavidin-biotin interaction as a model system [27,28,29], it remained to be determined whether DeepTip^TM^ probes could also show this excellent performance in low-intensity molecular recognition events, such as that found in the Pru p 3-phytosphingosine system.

Pru p 3 is the main allergen in peaches [30] and belongs to the lipid transfer protein (LTP) family. Its tertiary structure comprises four α-helices connected by short loops stabilized by four disulphide bridges [31], resulting in a molecular weight of 9 kDa. As is commonly found in the LTP family, Pru p 3 exhibits a hydrophobic cavity (or tunnel) [31] that can interact with a broad range of hydrophobic molecules in vitro. Phytosphingosine is a common lipid both in plants and in some animals and is a component of the natural ligand that interacts with the Pru p 3 through interactions of hydrophobic origin [32]. Since the hydrophobic interaction is one of the weakest interactions that can be established between molecules [33], the characterization of the Pru p 3-phytosphingosine system appears to be an adequate validation test to determine the performance of the proposed methodology for the characterization of these low-intensity molecular recognition events.

Following this rationale, the interaction between Pru p 3 and phytosphingosine was assessed with an atomic force microscope using DeepTip^TM^ probes and MicroDeck^TM^ substrates. Efficient crosslinking procedures were first developed to immobilise the phytosphingosine to the probes and the Pru p 3 to the substrates. Subsequently, single-molecular recognition events were identified from AFM force–distance curves. A high yield of over 70% successful events was found, as calculated from the ratio between the curves assigned to the specific interaction between protein and ligand, and the total ratio of recorded curves. This high success rate demonstrates the ability of this approach to be employed either in affinity atomic force microscopy studies or even in the force spectroscopy analysis of this interaction.

## 2. Materials and Methods

### 2.1. Materials

Recombinant Pru p 3 (rPru p 3) was produced in *Pichia pastoris* as described elsewhere [34], and purified by two consecutive chromatographic methods: exclusion chromatography and reverse-phase high-performance liquid chromatography (HPLC). Purity after the final HPLC step was assessed through SDS–polyacrylamide gel electrophoresis (SDS-PAGE) and mass spectrometry analysis. Commercial phytosphingosine was purchased from Cayman Chemical (Ann Arbor, Michigan, USA). Amine-functionalized DeepTip^TM^ SiO R11 atomic force microscopy (AFM) probes (elastic constant k = 0.01 N/m), and amine-functionalized MicroDeck^TM^ Si 150 substrates were kindly provided by Bioactive Surfaces S.L. (Galapagar, Madrid, Spain).

### 2.2. Thiol Modification of DeepTip^TM^ Probes and MicroDeck^TM^ Substrates

DeepTip^TM^ probes were incubated for 30 min in carbonate buffer at pH 9.1 and room temperature. After the removal of the carbonate buffer, the probes were incubated in 200 μL of a 2.5 mg/mL sulfo-LC-SPDP (sulfosuccinimidyl 6-[3′-(2-pyridyldithio)propionamido]hexanoate) solution in PBS-EDTA for 2 h at room temperature and covered with Parafilm to avoid evaporation. The probes were subsequently washed in PBS-EDTA three times for a duration of 2 min each time. In order to obtain reactive sulfhydryl groups on the surface, the probes were incubated with 200 μL of a 3 mg/mL solution of TCEP (Tris(2-carboxyethyl)phosphine) in PBS-EDTA for 15 min at room temperature. After the removal of the TCEP solution, the probes were washed three times with PBS-EDTA for a duration of 2 min each time.

MicroDeck^TM^ substrates were incubated for 30 min in carbonate buffer at pH 9.1. After the removal of the carbonate buffer, the substrates were incubated in 200 μL of a 2.5 mg/mL OPPS-PEG5K-SCM in PBS-EDTA solution for 1 h at room temperature and covered with Parafilm to avoid evaporation. The substrates were subsequently washed three times in a PBS-EDTA solution for 2 min each time. The obtaining of reactive sulfhydryl groups on the surfaces was performed using a TCEP solution and followed the same steps as described above for the probes.

The efficiency of this part of the experimental procedure was assessed by testing the presence of reactive sulfhydryls after incubation with the TCEP solution on the surface of the DeepTip^TM^ probes with the usage of the fluorophore 5-IAF (5-iodoacetamido-fluorescein) (Thermo Scientific), since it binds specifically to these reactive groups. Probes were incubated in 200 μL of a 500 μg/mL 5-IAF solution in DMF (dimethylformamide) for 30 min at room temperature. After the removal of the solution, the probes were washed in a 10% SDS solution in water for 5 min and, subsequently, with a PBS-EDTA solution three times with a duration of 5 min each time. Each probe was rinsed with mQ water (18 Mohm·cm). The difference in fluorescence between the samples incubated with 5-IAF and a non-functionalized control sample is shown in Supplementary Figure S1.

### 2.3. Decoration of the DeepTip^TM^ Probes with Phytosphingosine

The amine group present in phytosphingosine was used to covalently bind the molecule with the sulfo-LC-SPDP crosslinker. A quantity of 1 mg of phytosphingosine was dissolved in 1 mL of DMSO (dimethylsulfoxide) by heating the mixture in a thermoblock at 37 °C for 15 min with agitation. Subsequently, 1.66 mg of sulfo-LC-SPDP was added to the solution and allowed to react for two hours. Modified phytosphingosine was incubated with the thiol-modified probes overnight at 4 °C.

### 2.4. Modification of Pru p 3 with Sulfo-LC-SPDP and Decoration of the MicroDeck^TM^ Substrates

The Pru p 3 protein was reconstituted in PBS pH 7.4 to a final concentration of approximately 3 mg/mL. Of a 20 mM sulfo-LC-SPDP solution, 25 μL in mQ water was added to 200 μL of the protein solution and allowed to react for 1 h at room temperature. In order to remove the non-reacted crosslinker, the protein solution was filtered with a Zeba spin 0.5 mL 7 kDa filter column (Thermo Fisher, Waltham, MA, USA). The efficiency of the Pru p 3 modification with sulfo-LC-SPDP is presented in Supplementary Table S1.

In order to decorate the MicroDeck^TM^ substrate, the modified protein was dissolved in PBS-EDTA at a concentration of 15 μg/mL, and 200 mL was added on the surface and allowed to react overnight at 4 °C covered with Parafilm to prevent the evaporation of the solvent.

### 2.5. Determination of the Pru p 3-phytosphingosine Force–Distance (F-d) Curves

Force–distance curves obtained through the interaction of phytosphingosine-decorated probes and the Pru p 3-decorated substrates were recorded at room temperature in PBS pH 7.4 with a Nanolife atomic force microscope (Nanotec S.L., Cantos, Spain) operated in the lithography mode with WSxM software [35]. Two hundred forty curves were produced with the following acquisition parameters: approach velocity 1000 nm/s, retraction velocity 500 nm/s, contact time 1 s and contact force 600–800 pN. In addition to the intermediate controls regarding specific aspects of the procedure indicated above, the number and quality of the recorded force–distance curves were taken as the main evidence for the validity of the complete experimental design.

## 3. Results and Discussion

### 3.1. Classification of the Force–Distance (F-d) Curves

**Figure 1:**
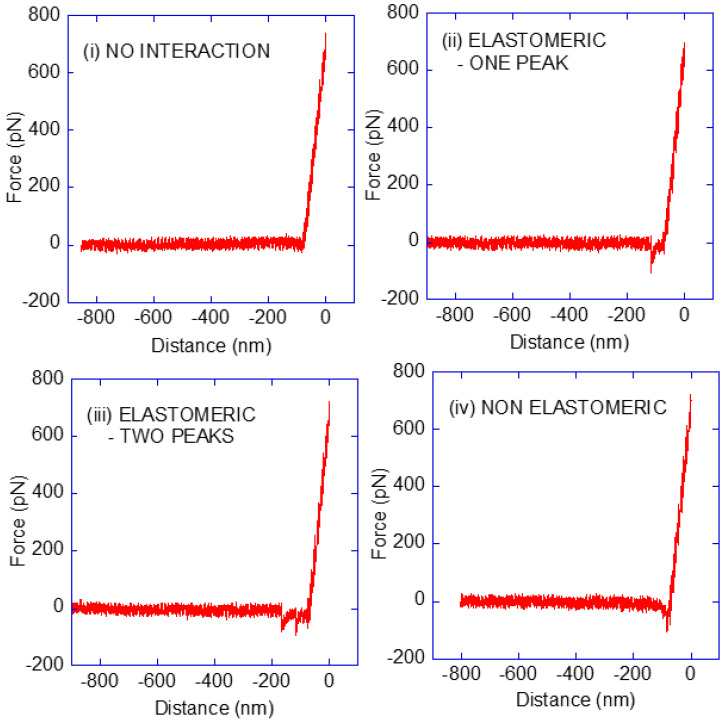
Classification of the force–distance curves obtained from the interaction between Pru p 3 and phytosphingosine. The curves were classified into four types: (**i**) no-interaction, (**ii**) elastomeric curve, (**iii**) elastomeric curve with two peaks, and (**iv**) non-elastomeric curve. Additionally, a small percentage of the curves (<2%) were discarded as experimental artefacts.

The force–distance curves obtained from the interaction between the Pru p 3 immobilized to the MicroDeck^TM^ substrate and the phytosphingosine immobilized to the DeepTip^TM^ AFM probe were classified into four types, as illustrated in Figure 1.

The classification of the curves follows three criteria: (i) the value of the adhesion force, (ii) the presence of one or two adhesion peaks in the curve, and (iii) the elastomeric or non-elastomeric character of the F-d curve in the adhesion region. In this regard, only adhesion forces >50 pN were considered indicative of a specific Pru p 3-phytosphingosine interaction event. In addition, the elastomeric character of the curve, which is commonly described as the tensile behaviour of a rubber (or elastic) band, implies a monotonous growth in the slope of the force–distance curve with increasing distance [36]. In this case, this elastomeric behaviour is assumed to correspond to the unfolding of the spacer in the OPPS-PEG5K-SCM molecule.

**Figure 2:**
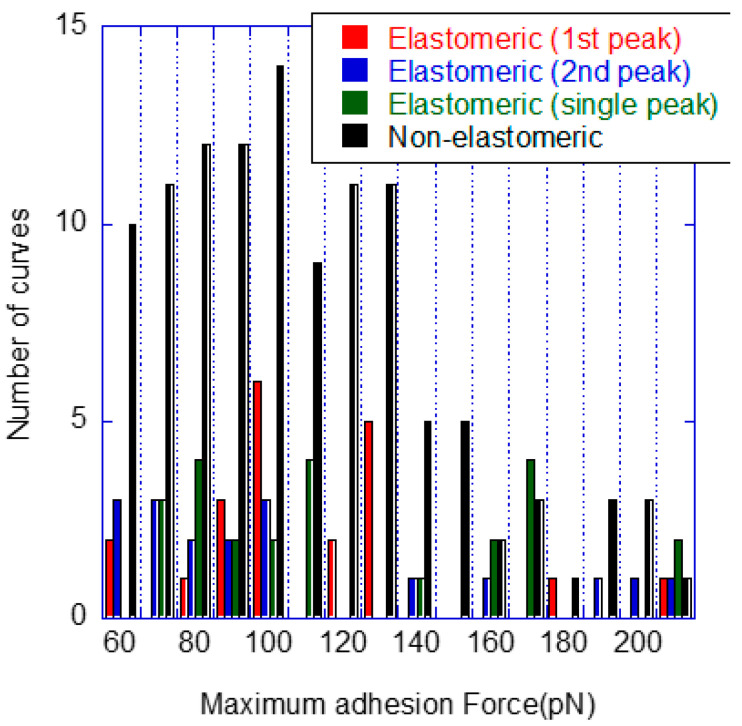
Distribution of the force–distance curves with respect to the absolute value of the maximum adhesion force recorded in the curve. The maximum force attained in the first and second peaks of the elastomeric–two-peak type are considered separately.

The distribution of curves following this classification in terms of the maximum adhesion force recorded from the interaction event is shown in Figure 2, in which it is distinguished between the peak measured in the first and second peaks of the elastomeric–two-peak curves.

**Figure 3:**
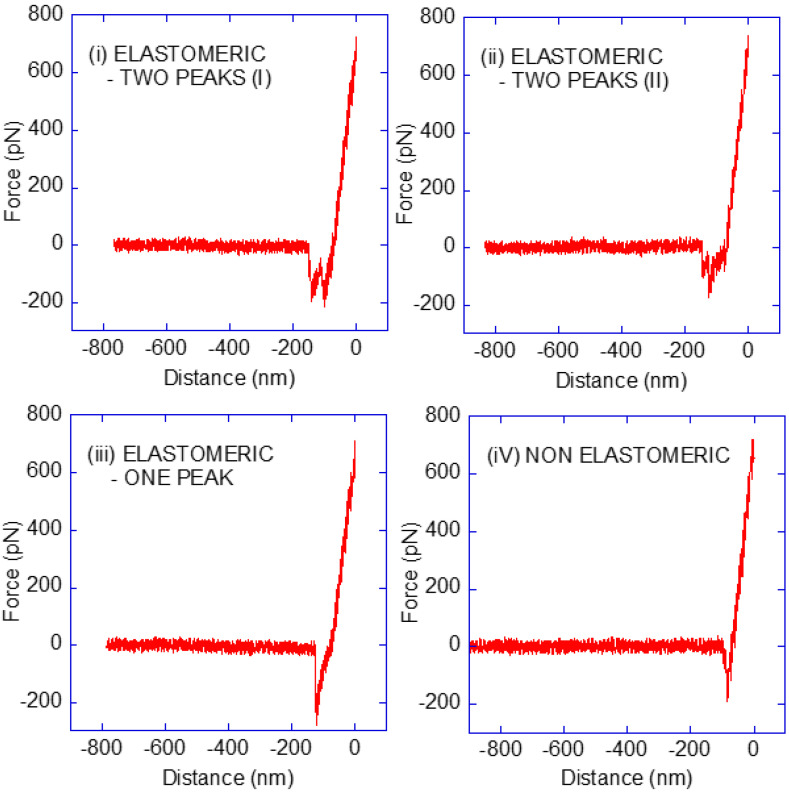
Representative examples of curves with high (>150 pN) values of the adhesion force. The two curves presented for the elastomeric–two-peak groups illustrate the observed trends: (**i**) curves with peaks of comparable adhesion force, and (**ii**) curves with a second adhesion peak with a significantly lower adhesion force. One curve is shown for (**iii**) the elastomeric–one-peak group and another representative curve (**iv**) for the non-elastomeric group.

The data shown in Figure 2 allows the identification of a clear drop in the number of curves at a force of approx. 150 pN. Consequently, the force–distance curves were further labelled as regular force or high force within each type, establishing the limit between both regimes at F = 150 pN. Some representative examples of high-force curves are presented in Figure 3.

**Figure 4:**
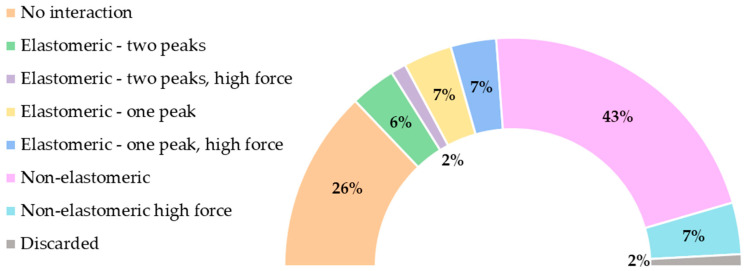
Summary of the distribution of the force–distance curves in each group distinguishing between regular and high adhesion forces. A curve was assigned to the elastomeric–two peaks group with high force if at least one of the peaks corresponded to an adhesion force of F > 150 pN. (Percentages: 26%—no interaction; 6%—elastomeric–two-peak; 2%; elastomeric–two-peak, high force; 7%—elastomeric–one-peak; 7% elastomeric–one-peak, high force; 43%—non-elastomeric; 7%—non-elastomeric, high-force; 2%—discarded).

The number of curves in each group—including the distinction between regular- and high-force curves—is summarized in Figure 4.

### 3.2. Interpretation of the Force–Distance (F-d) Curves

The analysis of the force–distance curves that result from the interaction of one target and one sensor molecules allows the obtaining of a deep insight into the system of interest at a molecular level from two complementary perspectives: On the one hand, it allows measuringthe intensity of the interaction established between both molecules, leading to the so-called force spectroscopy approach [37]. On the other hand, it may be used to identify the presence of the target molecule on a given surface, which may be a cell membrane, leading to the definition of affinity atomic force microscopy (A-AFM) [16]. The present work is mainly focused on this latter perspective and, consequently, on the determination of which curves may be considered a fingerprint of the interaction of interest, in this case, the interaction between the Pru p 3 protein and its ligand, phytosphingosine.

In accordance with the objective of this work, it is essential to establish the difference between a genuine sensor molecule–target molecule interaction and a nonspecific interaction not related to the identification of the molecule of interest. It is worth indicating that there are a few prerequisites to be fulfilled before undertaking the detailed analysis of proper and nonspecific interactions.

A first precondition is the availability of a sufficient number of force–distance curves of enough quality. Ideally, these curves should only show a single or a reduced number of peaks, while the rest of the curve should be clearly assigned to either the contact between the tip and the substrate (typically a straight segment at the right side of the curve, as illustrated in Figure 1 and Figure 3) or to the absence of interaction between both elements at higher distances (a horizontal line at the left side of the curve, as illustrated in Figure 1 and Figure 3). In this regard, the combination of the DeepTip^TM^ probes and MicroDeck^TM^ substrates appears as an efficient option, since the number of discarded curves is lower than 2% of the total number of curves.

A second precondition is the recording of curves that do not reflect any interaction (Figure 1, no interaction) distributed along the experiment. Firstly, the absence of this type of curve might reflect the nonspecific interaction of the phytosphingosine ligand with the substrate. In addition, their recurrent appearance during the experiment also precludes the possibility of possible contamination of the probe that might lead to measuring purely nonspecific interactions. In particular, the no-interaction curve shown in Figure 1 corresponds to curve 235, out of a total of 240 curves, which indicates that no systematic nonspecific interaction has affected the recorded data up to that moment, either as the result of a nonspecific interaction between the phytosphingosine and the substrate or of a subsequent contamination of the probe.

From the set of valid curves that reflect some kind of interaction between the probe and the substrate, it is necessary to identify those curves that originated from the specific interaction between the sensor molecule and the target molecule, in contrast to those that might originate from a spurious interaction. At this point, the usage of the OPPS-PEG5K-SCM crosslinker, to covalently bind the Pru p 3 to the substrate, has the advantage of offering a fingerprint for the specific interaction between the protein and phytosphingosine. This fingerprint arises from the PEG5K spacer, since the unfolding of this spacer during the retraction of the AFM tip from the substrate leaves the characteristic mark of an elastomer in the force–distance curve. This mark corresponds to a monotonous increase of the (absolute) value of force with increasing distance, such that the slope of the F-d curve is always positive until the detachment of the sensor and target molecules. In effect, this elastomeric trait is clearly observed in the types elastomeric–one-peak and elastomeric–two-peak curves in Figure 1 and Figure 3. In addition, the theoretical fully extended length of the PEG5K spacer is approx. 30 nm, and this concurs with the mean value of the length of the peak in the force–distance curve obtained by considering either the single peak of the elastomeric–one-peak curves or either peak of the elastomeric–two-peak curves.

In this context, the existence of a significant number of curves with two elastomeric peaks provides strong support for the identification of these peaks as those reflecting the genuine Pru p 3-phytosphingosine interaction, since it may be considered highly unlikely that two nonspecific events may yield the same traits in terms of elastomeric stretching, maximum value of the adhesion force and length of the peak as those expected for the interaction of interest. Consequently, it may be concluded that all elastomeric–two-peak curves correspond to the successful recognition of Pru p 3 (considered the target molecule) by a phytosphingosine molecule (considered the sensor molecule). It may be argued that these elastomeric–two-peak curves may be the most promising candidates to inspect the details of these interactions, but, as indicated above, this analysis is beyond the scope of this work.

Taking the elastomeric–two-peak curve as the gold standard for the Pru p 3–phytosphingosine interaction, it is possible to conclude that the elastomeric–one-peak curves must also reflect a genuine sensor–target molecule interaction between these moieties, since the peaks found in these curves show the same features as those previously found in either peak of the elastomeric–two-peak curves: elastomeric stretching, similar maximum value of the adhesion force and similar value of the length of the peak.

The assignment of the non-elastomeric curves to either a genuine or to a spurious interaction requires some additional discussion. Thus, although the presence of a characteristic elastomeric stretching of the curve is a fundamental guide for the identification of the genuine interaction, its absence does not necessarily imply that the curve does not result from the recognition of a Pru p 3 protein by a phytosphingosine. For instance, some kind of initial orientation of both molecules might prevent the expected unfolding of the PEG5K, so that no mark of its presence is recorded in the curve. At this point, the analysis of the distribution of curves presented in Figure 2 indicates some statistical similarities between the distribution of curves corresponding to the non-elastomeric type and those of the elastomeric type (either with a single peak or with two peaks). This observation may be expressed quantitatively using the Test of Goodness of Fit [38]. Since the interpretation of the high-force curves will require a subsequent analysis, at this point, the comparison between non-elastomeric and elastomeric curves will be restricted to the regular values of the adhesion force.

In order to apply the Test of Goodness of Fit, the values of the adhesion force (the maximum value of the peak) were distributed in intervals of 10 pN from the lowest value of 60 pN to the highest value of 140 pN. Since the number of non-elastomeric curves was significantly higher than that of the elastomeric types, the probability of finding a curve in an interval for the non-elastomeric curves was calculated from the ratio between the number of curves in that interval and the total number of non-elastomeric curves. Subsequently, the Test of Goodness of Fit was applied to the three possible elastomeric peaks found in each type of elastomeric curve: single-elastomeric peak, first peak of a two-peak elastomeric curve, and second peak of an elastomeric curve. The values of X^2^ calculated from the experimental data for each group of peaks are shown in Table 1 and compared with the values of the χ^2^ distribution with eight degrees of freedom (number of intervals minus one) and a level of significance of 5%.

The data in Table 1 indicate the similarity of the distribution of peaks between the non-elastomeric and the elastomeric curves. In this regard, this statistical analysis supports the inclusion of the non-elastomeric curves among the successful recognition events between Pru p 3 and phytosphingosine, although the curves themselves are less neat than those assigned to the elastomeric types. In summary, it may be argued that the non-elastomeric curves correspond to genuine interaction events (in the framework of affinity atomic force microscopy), but their use for the quantitative characterization of the interaction (in the framework of force spectroscopy) may be doubtful.

Lastly, it is necessary to discuss the appearance of the high-adhesion force curves in all three types of curves, as illustrated in Figure 3. There are at least two possible mechanisms that can account for the appearance of these events: (1) the interaction between two Pru p 3 proteins and two phytosphingosine molecules that detach simultaneously, and (2) the existence of a preferred orientation in the molecule that leads to events of distinct energies, despite corresponding to a Pru p 3–phytosphingosine recognition event.

The first mechanism has been described in the streptavidin–biotin system, where high forces (in the range of 2000 pN) were identified in some force–distance curves and assigned to the simultaneous detachment of two streptavidin and two biotin molecules [24]. The existence of this first mechanism is supported by the elastomeric–two-peak observations (II) described in Figure 3, where it can be observed that the first peak is followed by a drop in force to approximately half of the maximum value and, in addition, the elastomeric trait is found in the curve between the first and the second peaks.

However, there is an indication that some high-force events may not be related to this simultaneous detachment mechanism. Thus, in the elastomeric–two-peak observation (I), the presence of two high-force maxima can be observed. From the point of view of the simultaneous detachment mechanisms, this curve would correspond to the consecutive detachment of a pair of double Pru p 3-phytosphingosine interactions, such that each pair detached simultaneously. Although this event may not be impossible, it is doubtful that it may occur with enough frequency as to be observed from a sample of 240 curves.

**Figure 5:**
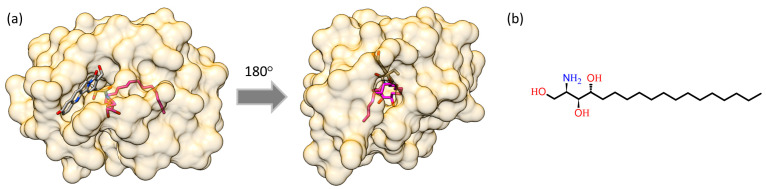
Model structure of the Pru p 3 with its native ligand. (**a**) The structure of Pru p 3 is depicted as a transparent surface, while the phytosphingosine structure is shown in stick representation. It is shown that the sphingosine tail (in pink sticks) is localized within the hydrophobic tunnel of Pru p 3. Two orientations are displayed after a 180-degree rotation to illustrate the openings of the hydrophobic tunnel. Images were rendered using UCSF Chimera-1,17.3-win64 software. (**b**) Structural formula of phytosphingosine.

In order to understand the possible origin of the second mechanism, it is necessary to consider the tertiary structure of the Pru p 3 and the possible geometry of its interaction with the phytosphingosine molecule. The structure of the Pru p 3 complex with its ligand was obtained from the results of the article by Cubells-Baeza et al. [32]. Phytosphingosine alone was docked into the hydrophobic cavity of the crystal structure of Pru p 3 with PDB code 2ALG [39] using AutoDock Vina calculations [40]. Subsequently, the resulting structure underwent a 10 ns molecular dynamics (MD) simulation. The 3D structure of the Pru p 3 is shown in Figure 5a and the structural formula of phytosphingosine is shown in Figure 5b.

In Figure 5, it is shown how the phytosphingosine tail interacts with Pru p 3, being localized within the hydrophobic tunnel of the protein. The geometry of this tunnel has led to the proposal of alternative spatial orientations that would account for the interaction between the protein and its ligand [41], such that each orientation corresponds to a different minimum in the energy landscape. In this context, it may be hypothesized that the difference between regular- and high-adhesion forces might reflect, in some cases, the different orientation of the ligand with respect to the protein. The significant proportion of high-force adhesion events, however, does not preclude that both proposed mechanisms might be acting simultaneously. In any case, and from the point of view of affinity atomic force microscopy, it can be argued that, independently from its detailed origin, high-adhesion force events also correspond to the genuine interaction between Pru p 3 and its ligand.

### 3.3. New Perspectives Opened by High-Yield A-AFM

After the previous discussion, it may be worth commenting on the possibilities offered by AFM with regard to the characterization of biomimetic and biological systems when a sufficiently high-yield regime is reached. In this regard, this particular application of AFM has been largely influenced by a success rate, defined as the number of curves that contain information on the specific interaction with respect to the total number of curves, typically in the range of 1% or even lower [25,26]. One of the main consequences of this relatively low success rate is the large number of curves required for the characterization of a given system, typically in the range of 10,000 curves or more. In addition to the associated time consumption, this procedure also limits the efficient obtaining of images in which the presence of the target molecule on the surface can be mapped. Consequently, it is a common practice to restrict the information recorded from a system to the retrieved force–distance curves and their subsequent analysis [42,43,44]. Although AFM offers the possibility of producing this type of mapping with the use of the Tip-Enhanced Raman Spectroscopy (TERS) technique [45,46], this latter approach does not allow an easy measurement of the intensity of the interaction between the sensor and target molecules. In this context, even a success rate of at least 10% (significantly lower than the one obtained with the procedure used in this work) would imply that a number of curves in the range of approximately 10 might be employed to establish or discard the presence of the target on a given area with sufficient confidence. Both the time required to obtain this number of force–distance curves, as well as the possibility of decorating the AFM probe with a large range of sensor molecules, are clear hints of the feasibility of this approach.

## 4. Conclusions

The usage of AFM-derived techniques (affinity atomic force spectroscopy and force spectroscopy) for the characterization of the single-molecular interaction between the lipid transfer protein Pru p 3 and phytosphingosine following a robust and versatile procedure is established in this work. Immobilization procedures for the covalent attachment of the sensor molecule (phytosphingosine) to the DeepTip^TM^ probe, and of the target molecule (Pru p 3) to the MicroDeck^TM^ substrate, were developed, so that the interaction between both molecules can be characterized from force–distance curves. The force–distance curves were recorded, and we concluded that over 70% of these curves correspond to the successful recognition of the target molecule by the sensor molecule and, consequently, can be used in the context of affinity atomic force microscopy measurements. Among all the successful events, a percentage close to 20% is also considered to be adequate to proceed with the analysis of the system for a force spectroscopy analysis. In summary, the successful application of this strategy to the characterization of the low-intensity hydrophobic interaction proper of the Pru p 3-phytosphingosine system is a clear indication of the potential offered by this experimental approach to assess an extremely wide range of different molecular recognition events of interest.

## Figures and Tables

**Table 1:** Test of Goodness of Fit applied to the peaks recorded from elastomeric–one-peak curves and the first and second peaks of the elastomeric–two-peak curves, taking the distribution of the value of adhesion forces in non-elastomeric curves as reference.

Curve	X^2^	χ^2^_0.05_ (f = 9)
Single elastomeric	12.2	15.51
First peak —two-peak elastomeric	13.2	
Second peak —two-peak elastomeric	2.7	

## Data Availability

The data presented in this study are available on request from the corresponding author.

## Supplementary Materials

The following supporting information can be downloaded at: https://www.mdpi.com/article/10.3390/biomimetics8080595/s1, Figure S1: Assessment of the presence of reactive sulfhydryl groups on the surface of the DeepTip^TM^ probes; Table S1: assessment of the modification of Pru p 3 with sulfo-LC-SPDP.

## References

[1] Schmitt O.H. Some interesting and useful biomimetic transforms. Proceedings of the Third International Biophysics Congress.

[2] Mirvakili S.M., Hunter I.W. (2018). Artificial Muscles: Mechanisms, Applications, and Challenges. Adv. Mater..

[3] Rodriguez N.R.M., Das S., Kaufman Y., Israelachvili J.N., Waite J.H. (2015). Interfacial pH during mussel adhesive plaque formation. Biofouling.

[4] Binnig G., Quate C.F., Gerber C. (1986). Atomic Force Microscope. Phys. Rev. Lett..

[5] Badin R., Burgain J., Desobry S., Bhandari B., Prakash S., Gaiani C. (2022). Probing maltodextrins surface properties by atomic force microscopy: Interplay of glass transition and reconstitution properties. Food Hydrocoll..

[6] Horber J.K.H., Miles M.J. (2003). Scanning Probe Evolution in Biology. Science.

[7] Dufrêne Y.F., Ando T., Garcia R., Alsteens D., Martinez-Martin D., Engel A., Gerber C., Müller D.J. (2017). Imaging modes of atomic force microscopy for application in molecular and cell biology. Nat. Nanotechnol..

[8] Dumitru A.C., Conrard L., Giudice C.L., Henriet P., Veiga-Da-Cunha M., Derclaye S., Tyteca D., Alsteens D. (2018). High-resolution mapping and recognition of lipid domains using AFM with toxin-derivatized probes. Chem. Commun..

[9] Müller D.J., Dufrêne Y.F. (2011). Atomic force microscopy: A nanoscopic window on the cell surface. Trends Cell Biol..

[10] Daza R., Cruces J., Arroyo-Hernández M., Marí-Buyé N., De la Fuente M., Plaza G.R., Elices M., Pérez-Rigueiro J., Guinea G.V. (2015). Topographical and mechanical characterization of living eukaryotic cells on opaque substrates: Development of a general procedure and its application to the study of non-adherent lymphocytes. Phys. Biol..

[11] Frisbie C.D., Rozsnyai L.F., Noy A., Wrighton M.S., Lieber C.M. (1994). Functional Group Imaging by Chemical Force Microscopy. Science.

[12] Dammer U., Hegner M., Anselmetti D., Wagner P., Dreier M., Huber W., Güntherodt H. (1996). Specific antigen/antibody interactions measured by force microscopy. Biophys. J..

[13] Riener C.K., Stroh C.M., Ebner A., Klampfl C., Gall A.A., Romanin C., Lyubchenko Y.L., Hinterdorfer P., Gruber H.J. (2003). Simple test system for single molecule recognition force microscopy. Anal. Chim. Acta.

[14] Kienberger F., Ebner A., Gruber H.J., Hinterdorfer P. (2005). Molecular Recognition Imaging and Force Spectroscopy of Single Biomolecules. Accounts Chem. Res..

[15] Yu H., Siewny M.G.W., Edwards D.T., Sanders A.W., Perkins T.T. (2017). Hidden dynamics in the unfolding of individual bacteriorhodopsin proteins. Science.

[16] Grandbois M., Dettmann W., Benoit M., Gaub H.E. (2000). Affinity Imaging of Red Blood Cells Using an Atomic Force Microscope. J. Histochem. Cytochem..

[17] Dumitru A.C., Herruzo E.T., Rausell E., Ceña V., Garcia R. (2015). Unbinding forces and energies between a siRNA molecule and a dendrimer measured by force spectroscopy. Nanoscale.

[18] Barattin R., Voyer N. (2008). Chemical modifications of AFM tips for the study of molecular recognition events. Chem. Commun..

[19] Volcke C., Gandhiraman R.P., Gubala V., Doyle C., Fonder G., Thiry P.A., Cafolla A.A., James B., Williams D.E. (2010). Plasma functionalization of AFM tips for measurement of chemical interactions. J. Colloid Interface Sci..

[20] Bergkvist M., Cady N.C., Mark S.S. (2011). Chemical Functionalization and Bioconjugation Strategies for Atomic Force Microscope Cantilevers. Bioconjugation Protocols: Strategies and Methods.

[21] Wildling L., Unterauer B., Zhu R., Rupprecht A., Haselgrübler T., Rankl C., Ebner A., Vater D., Pollheimer P., Pohl E.E. (2011). Linking of Sensor Molecules with Amino Groups to Amino-Functionalized AFM Tips. Bioconjug. Chem..

[22] Ebner A., Hinterdorfer P., Gruber H.J. (2007). Comparison of different aminofunctionalization strategies for attachment of single antibodies to AFM cantilevers. Ultramicroscopy.

[23] Bioactive Surfaces S.L. www.bioactivesurfaces.com.

[24] Corregidor D., Tabraue R., Colchero L., Daza R., Elices M., Guinea G.V., Pérez-Rigueiro J. (2023). High-Yield Characterization of Single Molecule Interactions with DeepTip^TM^ Atomic Force Microscopy Probes. Molecules.

[25] Guo S., Ray C., Kirkpatrick A., Lad N., Akhremitchev B.B. (2008). Effects of Multiple-Bond Ruptures on Kinetic Parameters Extracted from Force Spectroscopy Measurements: Revisiting Biotin-Streptavidin Interactions. Biophys. J..

[26] Sedlak S.M., Schendel L.C., Gaub H.E., Bernardi R.C. (2020). Streptavidin/biotin: Tethering geometry defines unbinding mechanics. Sci. Adv..

[27] Wong J., Chilkoti A., Moy V.T. (1999). Direct force measurements of the streptavidin–biotin interaction. Biomol. Eng..

[28] Yuan C., Chen A., Kolb P., Moy V.T. (2000). Energy Landscape of Streptavidin−Biotin Complexes Measured by Atomic Force Microscopy. Biochemistry.

[29] Teulon J.-M., Delcuze Y., Odorico M., Chen S.-w.W., Parot P., Pellequer J.-L. (2011). Single and multiple bonds in (strept)avidin-biotin interactions. J. Mol. Recognit..

[30] Missaoui K., Gonzalez-Klein Z., Pazos-Castro D., Hernandez-Ramirez G., Garrido-Arandia M., Brini F., Diaz-Perales A., Tome-Amat J. (2022). Plant non-specific lipid transfer proteins: An overview. Plant Physiol. Biochem..

[31] Gonzalez-Klein Z., Cuevas-Zuviria B., Wangorsch A., Hernandez-Ramirez G., Pazos-Castro D., Oeo-Santos C., Romero-Sahagun A., Pacios L.F., Tome-Amat J., Scheurer S. (2021). The key to the allergenicity of lipid transfer protein (LTP) ligands: A structural characterization. Biochim. et Biophys. Acta (BBA) Mol. Cell Biol. Lipids.

[32] Cubells-Baeza N., Gómez-Casado C., Tordesillas L., Ramírez-Castillejo C., Garrido-Arandia M., González-Melendi P., Herrero M., Pacios L.F., Díaz-Perales A. (2017). Identification of the ligand of Pru p 3, a peach LTP. Plant Mol. Biol..

[33] Israelachvili J.N. (2011). Intermolecular and Surface Forces.

[34] Pazos-Castro D., Gonzalez-Klein Z., Montalvo A.Y., Hernandez-Ramirez G., Romero-Sahagun A., Esteban V., Garrido-Arandia M., Tome-Amat J., Diaz-Perales A. (2022). NLRP3 priming due to skin damage precedes LTP allergic sensitization in a mouse model. Sci. Rep..

[35] Horcas I., Fernández R., Gómez-Rodriguez J.M., Colchero J., Gomez-Herrero J., Baro A.M. (2007). WSXM: A software for scanning probe microscopy and a tool for nanotechnology. Rev. Sci. Instrum..

[36] Pérez-Rigueiro J. (2023). Biological Materials and Biomaterials.

[37] Yang B., Liu Z., Liu H., Nash M.A. (2020). Next Generation Methods for Single-Molecule Force Spectroscopy on Polyproteins and Receptor-Ligand Complexes. Front. Mol. Biosci..

[38] Brandt S. (1998). Statistical and Computational Methods in Data Analysis.

[39] Pasquato N., Berni R., Folli C., Folloni S., Cianci M., Pantano S., Helliwell J.R., Zanotti G. (2006). Crystal Structure of Peach Pru p 3, the Prototypic Member of the Family of Plant Non-specific Lipid Transfer Protein Pan-allergens. J. Mol. Biol..

[40] Trott O., Olson A.J. (2010). Software News and Update AutoDock Vina: Improving the speed and accuracy of docking with a new scoring function, efficient optimization, and multithreading. J. Comput. Chem..

[41] Cuevas-Zuviría B., Garrido-Arandia M., Díaz-Perales A., Pacios L.F. (2019). Energy Landscapes of Ligand Motion Inside the Tunnel-Like Cavity of Lipid Transfer Proteins: The Case of the Pru p 3 Allergen. Int. J. Mol. Sci..

[42] Velázquez-Carreras D., Gavilan-Herrera M., Martinez-Martin I., Suay-Corredera C., Dumitru A.C., Galán E.H., Alegre-Cebollada J. (2023). Towards a new modular polyprotein system compatible with single-molecule force spectroscopy by atomic force microscopy and magnetic tweezers. Biophys. J..

[43] Milles L.F., Schulten K., Gaub H.E., Bernardi R.C. (2018). Molecular mechanism of extreme mechanostability in a pathogen adhesin. Science.

[44] Rico F., Russek A., González L., Grubmüller H., Scheuring S. (2019). Heterogeneous and rate-dependent streptavidin–biotin unbinding revealed by high-speed force spectroscopy and atomistic simulations. Proc. Natl. Acad. Sci. USA.

[45] Verma P. (2017). Tip-Enhanced Raman Spectroscopy: Technique and Recent Advances. Chem. Rev..

[46] Zhang Z., Sheng S., Wang R., Sun M. (2016). Tip-Enhanced Raman Spectroscopy. Anal. Chem..

