# Supplementary material for: Statistical Study of Low-Intensity Single-Molecule Recognition Events Using DeepTipTM Probes: Application to the Pru p 3-Phytosphingosine System
Source: Biomimetics (Basel). 2023 Dec 8;8(8):595. doi: 10.3390/biomimetics8080595 (PMC10742132; doi:10.3390/biomimetics8080595)
Supplement: Supplementary file 1 [file biomimetics-08-00595-s001.zip › biomimetics-2707781-supplementary.pdf]

biomimetics

MDPI

Supplementary Figure S1

Assessment of the presence of reactive sulfhydryl groups on the surface of the DeepTip™ probes with the usage of a fluorophore that specifically binds to these groups. (a) DeepTip™ probe without sulfo-LC-SPDP. (b) DeepTip™ probe incubated with sulfo-LC-SPDP and subsequent immersion in TCEP solution. Scale bar: 40 μm.

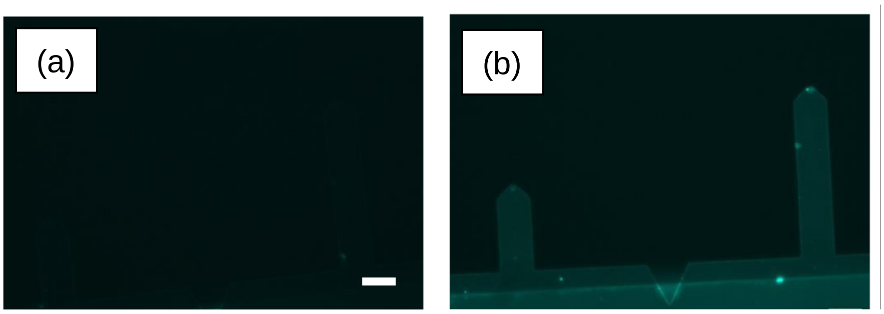

Supplementary Table S1

The assessment of the modification of Pru p 3 with sulfo-LC-SPDP was performed using a control sample prepared by mixing 2 μL of the modified protein solution after being filtered with 2 μL of PBS for 5 min. Alternatively, another sample with 2 μL of the modified protein solution after being filtered was mixed with 2 μl of a 3 mg/mL TCEP solution in PBS and allowed to react for 5 min. Absorbance was measured at 280 nm to identify the presence of the protein and at 343 nm to identify the presence of pyridine 2-thione. The pyridine 2-thione results from the reduction of the sulfo-LC-SPDP molecule.

Sample	Redox state	Abs 280 nm	Abs 343 nm
Control	Oxidized	0.064	1. 0.006
TCEP	Reduced	0.066	2. 0.047

Disclaimer/Publisher's Note: The statements, opinions and data contained in all publications are solely those of the individual author(s) and contributor(s) and not of MDPI and/or the editor(s). MDPI and/or the editor(s) disclaim responsibility for any injury to people or property resulting from any ideas, methods, instructions or products referred to in the content.
